# Virulence-Associated Secretion in *Mycobacterium abscessus*

**DOI:** 10.3389/fimmu.2022.938895

**Published:** 2022-07-08

**Authors:** Michal Bar-Oz, Michal Meir, Daniel Barkan

**Affiliations:** ^1^ Koret School of Veterinary Medicine, The Robert H. Smith Faculty of Agriculture, Food and Environment, The Hebrew University of Jerusalem, Rehovot, Israel; ^2^ The Ruth Rappaport Children’s Hospital, Rambam Medical Center, Haifa, Israel

**Keywords:** mycobacteria, abscessus, secretion, virulence, macrophage

## Abstract

Non-tuberculous mycobacteria (NTM) are a heterogeneous group of originally environmental organi3sms, increasingly recognized as pathogens with rising prevalence worldwide. Knowledge of NTM’s mechanisms of virulence is lacking, as molecular research of these bacteria is challenging, sometimes more than that of M. tuberculosis (Mtb), and far less resources are allocated to their investigation. While some of the virulence mechanisms are common to several mycobacteria including Mtb, others NTM species-specific. Among NTMs, Mycobacterium abscessus (Mabs) causes some of the most severe and difficult to treat infections, especially chronic pulmonary infections. Mabs survives and proliferates intracellularly by circumventing host defenses, using multiple mechanisms, many of which remain poorly characterized. Some of these immune-evasion mechanisms are also found in Mtb, including phagosome pore formation, inhibition of phagosome maturation, cytokine response interference and apoptosis delay. While much is known of the role of Mtb-secreted effector molecules in mediating the manipulation of the host response, far less is known of the secreted effector molecules in Mabs. In this review, we briefly summarize the knowledge of secreted effectors in Mtb (such as ESX secretion, SecA2, TAT and others), and draw the parallel pathways in Mabs. We also describe pathways that are unique to Mabs, differentiating it from Mtb. This review will assist researchers interested in virulence-associated secretion in Mabs by providing the knowledge base and framework for their studies.

## Introduction

The non-tuberculous mycobacteria (NTM) family is comprised of over 150 species, some of which are increasingly recognized as emerging pathogens, causing infections in immunocompromised as well as immunocompetent patients (1, 2). While most NTM are of only little pathogenic potential to humans, some – like *Mycobacterium marinum and Mycobacterium ulcerans*, are important pathogens, whereas others, like *Mycobacterium kansasii, Mycobacterium fortuitum* and *Mycobacterium abscessus* (Mabs), are considered opportunistic pathogens, but nevertheless can cause severe, often deadly, pulmonary infections (3–5). Mabs, classified as a rapidly growing mycobacterium (RGM), causes skin, lung and soft tissues infections that are very difficult to eradicate (6). Mabs is many times – but not strictly – an intracellular pathogen, and is found within phagocytic immune cells (macrophages and dendritic cells) and within epithelial and endothelial cells (7). To allow intracellular survival, these bacteria require unique mechanisms of resistance to the mostly hostile intracellular niche.

*Mycobacterium tuberculosis* (Mtb) employs various strategies to evade the immune response inside the infected cell: phagosome perforation and maturation arrest, cytokine-response downregulation, inhibition of reactive oxygen species (ROS) secretion, apoptosis prevention and more (8–11). These are dependent on different effector proteins, transported *via* specialized secretion systems that interrupt host means of defense in versatile manners (12). Although secretion systems and their respective substrates have been under thorough investigation in Mtb, much less is known of their counterparts in Mabs. Progress in bioinformatic methods, secretome analysis and high-throughput sequencing, combined with improved tools for transposon mutagenesis and clues drawn from Mtb studies, allowed the discovery of new effectors in Mabs. Recently, a considerable amount of effort has been made to assess the contribution of secreted proteins to the virulence of Mabs. Most studies employed deletion mutants and evaluation of bacterial proliferation in *ex-vivo* and *in-vivo* models of infection. In this review, we follow the route of infection in Mtb, introducing the secreted effector proteins known to affect Mtb virulence, and then comparing them to their analogs in Mabs. This approach, although may miss completely novel effectors in Mabs (that are fundamentally different from those of Mtb), will provide the reader with a solid starting point in assessing these factors in Mabs. In addition, we outline the gaps of knowledge regarding factors yet to be identified in Mabs – gaps the filling of which will probably require more complicated analyses of the Mabs’s secretome and proteome, as well as in-depth bioinformatics look at the bacterial genome.

## Secretion Systems in Mycobacteria

Secretion systems play an essential role in promoting virulence in mycobacteria (13–16). These sophisticated apparati enable the export of effector proteins across the thick membrane into the host cell, reducing immune response and promoting bacterial survival.

Three groups of secretion systems were identified in mycobacteria:

◼ Type VII secretion systems (T7SS): Known as ESX secretion systems, these were named after the 6kDa Early Secretory Antigenic Target (ESAT-6). Mtb’s ESAT-6 was established as a critical contributor to virulence, along with a second ESX-1 substrate CFP-10 (17). Partial deletion of the ESX-1 system accounts for the attenuation observed in BCG bacteria compared to their ancestor *M. bovis* (18, 19). The five different mycobacterial ESX systems (ESX-1-5) are encoded by paralogous loci that are widespread among the slow and rapidly growing species (20). In Mtb, ESX-1, ESX-3 and ESX-5 are critical components in virulence, while the roles of ESX-2 and ESX-4 remain obscure (21–24). Mabs, however, has only two identified ESX clusters: ESX-3, an essential secretion system involved in iron and zinc homeostasis; and ESX-4, recently shown to play a significant role in virulence, serving as an analogue for Mtb’s ESX-1[ (25, 26)].
◼ The Twin-Arginine Translocation (TAT) pathway: distinguished by the ability to transport polypeptides in their folded state. Substrates utilizing the TAT export system must hold a highly conserved twin-arginine leader motif (S/TRRXFLK) which is found in the N terminus of the protein (27, 28).
◼ The Sec pathway: The substrates exported across the cytoplasmic membrane by Sec systems are initially produced as precursor proteins, consisting of conserved amino- terminal signal sequences (29). During translocation, the signal peptide is cleaved to generate the mature exported protein. Two non-redundant Sec systems are identified in Mtb: SecA1 – an essential “housekeeping” system, and the accessory secretion factor - SecA2 (30, 31).

Generally, effector proteins secreted by mycobacteria each utilize a specific mechanism of export, depending on their individual traits.

Here, we will describe the main approaches Mtb uses to resist host cell’s antibacterial attempts, trying to unravel the analogous effectors in *Mabs*.

## Phagosome Permeabilization

As mycobacteria are inhaled into the lungs and reach the alveolar cavity, they are phagocytized by alveolar macrophages (32). The bacilli are enclosed in a vesicular phagosome which is next subjected to fusion with the lysosome, forming a mature phagosome (e.g phagolysosome) (33). Permeabilizing the phagosomal membrane with eventual phagosomal escape is a pivotal step in immune resistance, allowing the pathogen to secrete effectors into the cytosol. A crucial effector involved in phagosome rupture is the ESX-1 substrate EsxA/ESAT-6 (*Rv3875*), that creates a heterodimer with another secreted protein, EsxB/CFP-10 (*Rv3874*) (17). Mtb mutants defective in secretion of EsxA and/or EsxB fail to translocate to the cytosol and are attenuated, as seen in the vaccine strain *Mycobacterium bovis* BCG and in the H37Ra strain (19, 34–37). The mechanism in which EsxA contributes to phagosome membrane rupture is still a matter of debate, but it appears to induce gross membrane disruption in a contact-dependent manner (38, 39). As previously mentioned, Mabs does not possess an ESX-1 secretion system. However, work conducted by Laencina et al. (26) presented evidence of Mabs EsxT (*MAB_3753c*) and EsxU (*MAB_3754c*) serving as functional analogues to EsxA and EsxB, respectively, as their deletion reduces phagosome-to-cytosol contact. Moreover, rupture of the phagosomal membrane occurs only in the presence of an intact *eccB4* gene, a structural component of Mabs Esx-4 secretion system. Whether EsxT and EsxU directly damage the phagosome membrane is yet to be investigated.

## Phagosome Maturation and Acidification

After the phagosome internalizes the mycobacterium, a sequence of event initiates, which includes a decrease in pH and acquisition of antimicrobial properties. The late phagosome then fuses with the lysosome (Phagosome maturation), and the bacilli are processed into small particles, that are next presented to T-cells as antigens, initiating the adaptive immune response (40). Many mycobacterial secreted effectors target to disrupt this process.

Rab GTPases are molecular switches that coordinate the changes in phagosomal membrane upon internalizing the invader (41). Human Rab5 and Rab7 coordinate vesicle trafficking between the early phagosome to late endosome (Rab5) and from the late endosome to phagolysosome (Rab7) (42). Nucleoside diphosphate kinase A (NdkA, *Rv2445c*) is an Mtb GTPase secreted through the SecA2 pathway (30, 43, 44). NdkA binds Rab5 and Rab7 and facilitates the transition fromthe active GTP-bound state to the inactive GDP-bound state, interfering with the phagolysosome formation (45, 46). Also, NdkA inactivates Rac1 – a GTPase, required for activation of NADPH oxidase 2 (NOX2), thus blocking production of reactive oxygen species (ROS), and preventing bacterial killing (47). In Mabs the only annotated Ndk protein (*MAB_1606*) has an 86% similarity to Mtb’s NdkA, suggesting a similar role and function. Currently, no data on *MAB_1606* deletion in Mabs has been published, leaving the role of Ndk in Mabs unexplored.

The next step in phagosome maturation that is manipulated by Mtb is the production of lipid regulator phosphatidylinositol 3-phosphate (PI3P) from phosphatidylinositol (PI). PI3P is a membrane tag that signals the macrophages to continue down the phagolysosome biogenesis pathway (48). Also exported by SecA2 pathway in Mtb (30), secreted acid phosphatase M (SapM, *Rv3310*) dephosphorylates PI3P, limiting its ability to recruit PI3P-binding proteins in the mycobacterium-containing vacuole (MCV) membrane, and blocking the maturation process (49–51). A deletion mutant of SapM in Mtb is attenuated in human macrophages and *in-vivo* in guinea pigs (50, 52, 53). However, although found in other slow-growing nontuberculous mycobacteria such as *M. avium* and *M. marinum* (50, 54, 55), a SapM analog has not yet been identified in *M. abscessus*.

The mature phagosome assembly is mediated by many RabGTPase proteins, each necessary for to complete the intricate process of phagolysosome fusion. Human Rab7L1, in its active GTP-bound form Rab7L1-GTP is recruited to bacilli-containing phagosomes, and signals other phago-lysosomal markers such as the previously mentioned RAB7 (56). In Mtb, secreted protein kinase G (PknG) interacts with Rab7L1, limiting the formation of the active GTP-bound state and interfering with the successive signaling process (57–59). A *ΔpknG* mutant in Mtb is attenuated in mice after intravenous injections, but not by aerosol delivery (60). Though PknG in Mabs is completely uninvestigated, we can speculate with confidence that it performs in the same manner as in Mtb. A putative protein encoded by *MAB_4224* bears considerable similarity (82%) to Mtb’s PknG (*Rv0410c*). Furthermore, *MAB_4244* lies between the gene encoding the probable glutamine binding protein H (*glnH*, *MAB_4223*) and the gene for acetate kinase A (*ackA*, *MAB_4225c*), exactly like it does in Mtb (*glnH Rv0411c, ackA Rv0409*). The close downstream proximity of *pknG* to *glnH* is of importance, since these two genes are suggested to be co-expressed in a conserved operon in *Actinomycetes* (61, 62). With the lack of a well-established mechanism, it is thought that GlnH, through protein-protein interactions, activates PknG *via* a probable transmembrane protein, GlnX (61). All these suggest *Mabs* PknG may play an important yet under-explored role in Mabs pathogenesis.

Another Mtb effector found to reduce the recruitment of EEA1, Rab5 and Rab7 and therefore inhibit phagosome maturation, is TlyA (*Rv1694*) (63). TlyA serves as a ribosomal RNA methyl transferase (rRNase) (64) and displays an additional role as a hemolysin, when purified from Mtb and expressed in *M. smegmatis* (Msme) (38, 65). Rahman et al. (66) showed that TlyA forms oligomers on phagosome membrane and red blood cells, finally leading to lysis. Mtb TlyA Knockout demonstrated reduced growth in *ex-vivo* infected macrophages and in mouse models, yet the lack of complementation experiments precluded from drawing a definitive conclusion regarding its role and effect (67). Mtb TlyA resides in close proximity to RecN (Rv1696). *MAB_2359* is a putative rRNA methytransferase with 78% similarity to the Mtb’s TlyA, and is also located in proximity to Mabs recN, suggesting it is a TlyA analog, and that its role in pathogenesis should be explored further.

ESCRT (The host Endosomal Sorting Complexes Required for transport) pathway directs cargo destined for digestion in lysosomes, such as the Mtb-containing vacuole (MCV) (68). Moreover, ESCRT apparati facilitate antigen processing, therefore promoting T-cell activation during Mtb infection (69). The initial ESCRT machinery is assembled by several components, one of which is HRS (Hepatocyte growth factor-regulated tyrosine kinase substrate), a target for the Mtb Esx-3 secreted effector, EsxH (70). EsxH (Rv0288) forms a 1:1 heterodimer with another secreted molecule EsxG (Rv0287), together they serve as distinctly functional paralogues to the EsxA/EsxB complex in Mtb ESX-1 (71, 72). EsxH was demonstrated by co-immunoprecipitation assays to interact directly with HRS, diminishing the ESCRT assembly and inhibiting phago-lysosome fusion (70). Knock-down of HRS resulted in reduced MCV maturation, stressing the importance of this EsxH target. However, overexpression of Mtb’s EsxH in RAW cells had greater effect on phagosome maturation arrest than HRS depletion, suggesting that an additional target is aimed by EsxH (70). EsxH deletion caused considerable attenuation, with a 3-4 log decrease of CFU in the lungs of mice (69, 72) - however, some of this attenuation may be related to the role of ESX-3 in iron acquisition, rather than phagosome maturation arrest Among pathogenic mycobacteria, the Esx-3 cluster is highly conserved (73). Therefore, it is not unexpected to discover that Mabs EsxH (MAB_2228c) and EsxG (MAB_2229c) share relatively high similarity with their counterparts in Mtb (81% each). The contribution of Mabs ESX-3 secretion system to host responses was well-studied by Kim et el (25).. They created an Esx-3 deletion mutant (MAB_2224c-2234c, *Δesx-3*) to examine the effect on growth, inflammatory response and pathophysiology in *ex-* and *in-vivo* models. Although *in-vitro* growth of the mutant was not reduced, intracellular growth was significantly reduced in bone marrow derived macrophages (BMDM). Mice infected by the mutant exhibited lower bacillary loads 7 days post infection, but with no significant difference 14 days post infection. Also, mice infected by *Δesx-3* showed less severe lung pathology and decreased granulomatous infiltrates. Serum levels of TNF-α and IL-6, as well as mRNA levels of proinflammatory cytokines were also decreased in *Δesx-3* infected mice. A caveat of this study was the lack of complementation experiment, which make interpretation less straightforward. Additionally, since the ESX-3 secretion system is essential for maintaining homeostasis in an iron and zinc depleted environment, we cannot confidently attribute the consequences of its deletion to the absence of the EsxG/H complex and to it’s effect on phagosome maturation, from that on iron homeostasis. To do that would necessitate additional experiments which would attempt to separate the two functions of the ESX-3 system.

Acidification of the late phagosome is prompted by the presence of vacuolar ATPase (V-ATPase) proton pumps on the phagosomal membrane (74). One of the three phosphatases secreted by Mtb, protein tyrosine phosphatase A (PtpA, *Rv2234*), binds subunit H of V-ATPase (75, 76). However, the interaction between PtpA and subunit H was found to be through protein-protein interaction, suggesting that V-ATPase is not the catalytic substrate of PtpA (75). Rather, the interaction of PtpA with V-ATPase is a prerequisite for the main purpose of PtpA – dephosphorylating, and as a result inactivating, vacuolar protein sorting 33B (VPS33B) (75, 77). VPS33B, a member of ESCRT machinery, is involved in vesicle trafficking and responsible for the necessary alterations of membranes to promote phago-lysosome fusion. Bach et al. (77)showed direct binding and co-localization of PtpA and VPS33B in the cytosol and impaired recruitment of VPS33B to the lysosome in Mtb-infected macrophages. Mtb PtpA knockout (Δ*ptpA*) fails to inhibit phagosome acidification and maturation in human THP-1 macrophages (75, 77). Interestingly, Δ*ptpA* is attenuated for growth in *ex-vivo* infected macrophages, but not within *in-vivo* infected mouse model (78). However, this appears to be specific to the mouse model, and does not undermine the importance of PtpA in pathogenesis in humans. The putative protein encoded by *MAB_1900c* shares great similarity (81%) with PtpA in Mtb. The proximal gene *MAB_1901c* contains considerable similarity (75%) to *Rv2232*, the adjacent gene to *ptpA* in Mtb. *Rv2232* encodes for protein tyrosine kinase (PtkA), which phosphorylates and activates PtpA (77). Deleting *ptkA* in Mtb also leads to growth reduction in infected macrophages (79). The presence of this cluster in Mabs indicates an opportunity for further investigation of the contribution of Mabs- PtpA to virulence.

Another factor playing a role in blocking phagosome maturation of Mtb is lipoamide dehydrogenase C (LpdC, Rv0462). LpdC participates in the metabolism of branched-chain amino acids and is also secreted *via* the SecA2 pathway (30, 80). Its role in phagosome maturation inhibition was found as it demonstrated a cholesterol-dependent interaction with coronin-1 found on macrophages infected with Mtb and BCG (81, 82). MAB_4127c has an 88% similarity, and although some databases automatically annotated the gene as *lpdA*, it is probably the closest analog to the *lpdC* (*Rv0426*) from Mtb, whereas the true analog of Mtb’s LpdA, is MAB_3656c.

Overall, phagosome maturation seems to be a target for manipulation by Mabs, much like Mtb. The effectors involved in inhibiting this process are understudied in Mabs, and their investigation promises to be both fruitful and interesting.

## Autophagy Inhibition

Autophagy is a conserved degradation process of the cell in which unnecessary components and dysfunctional organelles are discarded in a regulated manner, allowing elimination of some and recycling of other materials through lysosome digestion (83). Proper and regulated autophagy is important for immune control of mycobacterial infections (84). Mycobacteria have several effectors aimed at disrupting this process. One anti-autophagy effector secreted by Mtb is the Enhanced Intracellular Survival (Eis, Rv2416c) protein (85). Mtb Eis has an N^ε^-acetyltransferase activity (86). Upon infection Eis acetylates DUSP16/MKP-7, a JNK-specific phosphatase therefore preventing DUSP16/MKP=7 from activating beclin1 (BCLN1), a necessary protein in autophagy regulation (86, 87). Eis is also proposed to inhibit autophagy through IL-10 upregulation (88), and through a JNK-dependent mechanism, affecting ROS production (89). An Mtb Eis deletion (Δ*eis*) causes increase in JNK activity, leading to elevated ROS and increased autophagy, proinflammatory response and host cell death. Nevertheless, Δ*eis* does not show reduced virulence in mice (89, 90). Mabs harbors two Eis encoding genes, Eis1 (*MAB_4124*), and Eis2 (*MAB_4532c*). Eis1 seems to be the closest homologue to Mtb’s Eis by Bidirectional Best Hit (BBH) search (91). However, it was shown that Mabs’ Eis1 does not modify aminoglycosides, and the basis for this lack of activity was pinpointed to structural reasons, where the active site is too narrow to accommodate large substrates like these antibiotics (92). Curiously, Mabs Eis2 deletion mutant (*Δeis2*) was dramatically attenuated in murine macrophages, showing increased ROS levels. Moreover, *Δeis2* was unable to penetrate the phagosomal membrane and induce phagosome-to-cytosol contact compared to WT Mabs (91).

## Reduction of Reactive Oxygen Species (ROS)

As previously mentioned, ROS are produced within phagosomes of infected macrophage as an additional antimicrobial ammunition. NADPH oxidase 2 (NOX2), the enzyme producing ROS, is a multiprotein complex that promotes a distinct phagosome maturation and autophagy-related pathway called LC-3 associated phagocytosis (LAP) (93). In the process of LAP, one membrane engulfs a pathogen, or pathogenic residues, instead of a double membrane in autophagy. Mtb **CpsA** (*Rv3484*) interferes with the recruitment of NOX2 to the phagosome and inhibits LAP through an unknown target (94). However, no clear analog in Mabs was found, and it is unclear if Mabs manipulates this pathway in the macrophage.

## Modulating Cytokine Response

Mycobacterial infection induces the secretion of a large number of cytokines, including interferon gamma (IFN-γ), interleukin-1 (IL-1), IL-2, IL-6, IL-10, IL12, IL-18 and tumor necrosis factor alpha (TNF-α) (11, 95). The immune response is provoked by some and downregulated by other cytokines to protect the host against unfavorable pathology. Mtb survival depends on its ability to subvert the immune response, in order to promote its proliferation and cell-to-cell spread. The following are mechanism of such immune-response and cytokine release modulation.

Enoyl coA hydratase A1 (EchA1, Rv0222) is secreted by Mtb through an unknown mechanism and reaches the host cell cytosol. Wang et al. found that EchA1 is subjected to ubiquitination by the host cell ubiquitin ligase ANAPC2 (96). Ubiquitinated EchA1 promotes the recruitment of SHP1 – a protein tyrosine phosphatase that interacts with TNF receptor-associated factor 6 (TRAF6) and inhibit its ubiquitination. Since ubiquitinated TRAF6 is a mediator in IL-1 signaling, its inhibition by Mtb EchA1 impairs the production of proinflammatory cytokines. Mice infected with Mtb EchA1 mutants produced much higher levels of IL-1b, IL-6 and IL-12 in lung tissue, and attenuation of *ΔechA1* growth was demonstrated in mice following aerosol infection (96). We found a probable enoyl coA hydratase gene in Mabs genome (*MAB_0606c*) with 44% similarity to Rv0222. One should note, though, that a 44% similarity is quite low, and take this homology with caution. No other information regarding this protein in Mabs is currently known.

Disulfide bond forming DsbE (also known as Mpt53, Rv2878c) is an effector that was found to activate protective host responses through promoting proinflammatory cytokine production. Predicted to be secreted by SecA1/2 (43, 97), DsbE binds, hence increases phosphorylation of TGF-β-activated kinase 1 (TAK1), an important signaling molecule downstream to TLR/TRAF6/TAB2 or TAB3 signaling pathway (98). Phosphorylated Tak1 activates the NF-kb pathway and the kinases JNK and p38, leading to biosynthesis of proinflammatory cytokines TNF, IL-6 and IL-12. An Mtb Δ*dsbE* mutant induces less TNF and IL-6 production in *ex-vivo* macrophage models and *in-vivo* in mice lungs (98). Moreover, Δ*dsbE* was hypervirulent in mice, with 1-2 log increase in CFU at 21 days post infection (98). It is therefore tempting to speculate whether Mabs probable DsbE (99), with relatively high resemblance to Mtb protein (78% similarity), stimulates similar responses.

## Cell Death Manipulation

Apoptosis and necrosis are two variants of cell death which greatly differ in all aspects, such as energy requirements, regulation, organelles fate and causes (100). Mtb produces effectors that favor the development of necrosis and inhibit the cell’s processes promoting apoptosis (101). Since the final consequence of many augmented immune response processes is delayed cell death, many effectors manipulate apoptosis indirectly. For example, Mtb protein tyrosine phosphatase B (PtpB, Rv0153c) is a broad-spectrum phosphatase that was shown to inhibit the IFN-γ-mediated activation of ERK1/2 and p38 signaling pathway, hence inducing production of IL-6 and inhibiting host cell death (102). However, no clear orthologue proteins to PtpB were described in Mabs. The closest protein found is MAB_4591, carrying a low similarity of 42%, which should be taken very cautiously.

Apoptosis is a redox-sensitive process. Mtb exploits this sensitivity and inhibits apoptosis by blocking ROS release by host cell into the phagosome. Superoxide dismutase A (SodA, Rv3846) is secreted by SecA2 and suspected to be involved in neutralization of superoxides produced by NOX2 in the MCV (31). Since SodA in an essential gene in Mtb, no deletion mutant could be established. However, a mutant with reduced SodA activity was constructed in Mtb and demonstrated *in-vitro* to be highly susceptible to killing by hydrogen superoxide (103). Moreover, mice infected by SodA-defective Mtb exhibited at least 10-fold more apoptotic cells in their lungs as compared to those infected by the WT strain (103). Mabs SodA (*MAB_0118c*) shares great similarity to its Mtb counterpart (89%). It will be intriguing to test whether Mabs mutants defective in SodA activity will have a phenotype consistent with the one in Mtb.

Mtb, as well as other pathogenic mycobacteria aspires to escape the intracellular niche in order to spread to other host cells. Encouraging necrosis is an effective approach to accomplish this goal (104). The outer membrane channel protein CpnT is required for efficient nutrient uptake in Mtb (105). While its N-terminal harbors a pore-forming capacity for this matter, its C-terminal domain, also called tuberculosis necrotizing toxin (TNT), can be released following proteolytic cleavage. TNT targets host cell coenzyme NAD^+^, thereby leading to its depletion (106). The necroptosis RIPK3/MLKL pathway is then activated, prompting the host cell death (107). Since deleting TNT does not attenuate bacilli growth *in-vivo*, it is suggested that Mtb has alternative pathways to promote necrosis (105). An analogous gene to Mtb CpnT/TNT analytic was not identified in the Mabs genome. However, a recent study found a cassette in a Mabs prophage with a polymorphic toxin (PT) that contains a C-terminal domain related to Mtb TNT (108). This finding offers a direction for further investigating CpnT/TNT analogues in Mabs, potentially involved in virulence.

## Discussion

**Figure 1:**
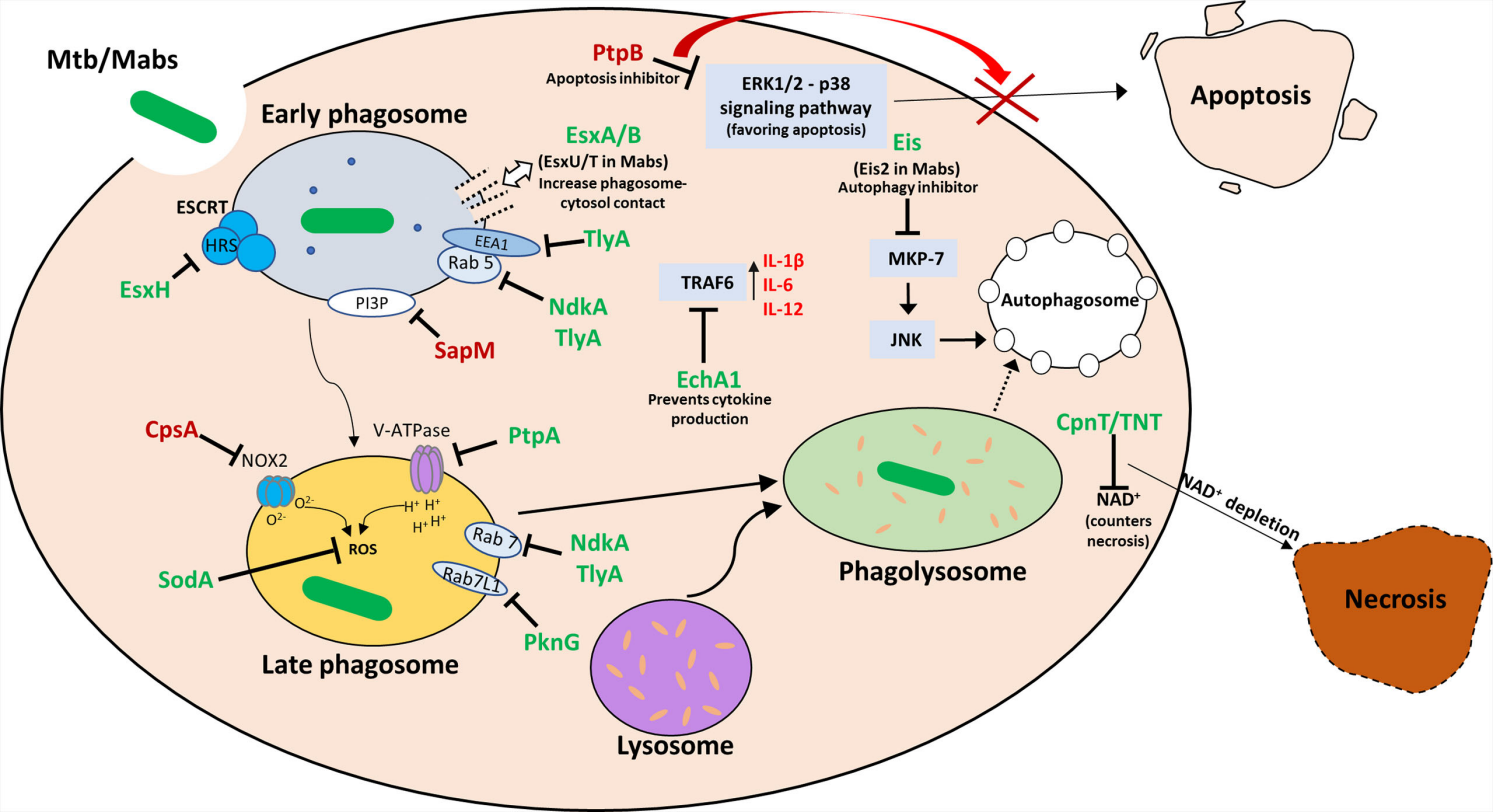
Visual summary of the secreted effectors in Mtb and Mabs and their intracellular targets. Once the bacilli are phagocytosed by the macrophage, it goes through a series of processing stages: early and late phagosome formation, phagosome acidification and phago-lysosome fusion. Necrosis is an undesirable outcome for the host, while it promotes cell-to-cell spread of the bacteria. Apoptosis, while leading to macrophage death, promotes effective immune response, and is therefore detrimental to the bacteria in the infection process. During each stage, the mycobacteria attempts to block phagosome maturation and acidification, prevent apoptosis and promote necrosis. Effectors are marked in green when a MABS analog of the Mtb protein is either identified or is presumed to exist, and in red when no MABS analog has been identified or suggested.

Like most intracellular pathogens, mycobacteria, while in the macrophage, secrete a myriad of effector molecules into the phagolysosome as well as the cytosol in order to counter the many mechanisms at the macrophage’s disposal aimed at destroying this very pathogen. Knowledge of these secreted effectors and their mechanisms of action can be implemented into novel therapeutics, construction of attenuated mutants for vaccine purposes and predictions of disease severity. Whereas in *M. tuberculosis* there has been considerable progress in the characterization of the compendium of secreted effectors, this is not the case in most non-tuberculous mycobacteria, including *M. abscessus*. Most known *M. abscessus*-secreted effectors are at least structurally related to well-characterized secreted effectors in *M. tuberculosis*. Novel, yet undiscovered effectors probably exist, and may be shared by other rapid-growing mycobacteria (such as *M. fortuitum*) – but as data on this is still scarce, it is difficult to provide specific examples. Additional data and research specifically into the pathogenesis of *M. abscessus* is needed to characterize such effectors. In this review we attempted to provide the “basic footwork” for researchers interested in exploring the secreted effectors of *M. abscessus* – by summarizing the knowledge on those effectors that have been, at least partially, characterized, and by providing the basic links connecting characterized *M. tuberculosis* effectors with genes and proteins in *M. abscessus*, that may – or may not – play a homologous role in this pathogen. These were also summarized in **Table 1**. **Figure 1** illustrates most of these effectors in a graphic manner. Obviously, this approach may miss completely novel and Mabs specific effectors that may very well exist - however their identification will necessitate more complicated experimental and bioinformatic analyses of the *M. abscessus* secretome, proteome and genome.

**Table 1:** Secreted effectors in *M. tuberculosis*, and their putative analogs in *M. abscessus*.

Name	Gene ID in Mtb	Secretion pathway	Protein function in Mtb	Host target	Host cell process	Impact of gene deletion on Mtb virulence	Gene ID Mabs	Identity / similarity
EsxA	Rv3875	ESX-1	?	TLR-2, SR-B1, B2M	Phagosome maturation	Attenuated ex-vivo and in vivo	MAB_3754c	No identity
Please note that whereas EsxA (ESAT-6) of Mtb is part of the ESX1 system, MAB_3754c is part of the ESX-4 system in MABS. An ESX-1 system does not exist in MABS. However, both system play what appears to be an analogous role in pathogenesis – hence the analogy we draw between EsxA and MAB_3754c, despite lack of biochemical identity.								
EsxH	Rv0288	ESX-3	Iron acquisition	HRS	Phagosome maturation	Attenuated ex vivo and in vivo	MAB_2228c	65% / 81%
SapM	Rv3310	SecA2	Phosphatase	Phosphatidyl-inositol3-phosphate	Phagosome maturation	Attenuated ex vivo and in vivo (guinea pig)	?	–
PknG	Rv0410c	SecA2	Serine/Threonine kinase	Rab7L1/Rab29	phagosome maturation	Attenuated ex vivo and in vivo	MAB_4224	73% / 82%
CpsA	Rv3484	?	Contains LCP and LytR domains	Inhibits NOX2 activation	phagosome maturation, ROS production	Attenuated ex vivo and in vivo (mouse and zebrafish model)	?	–
TlyA	Rv1694	?	rRNA methylase, hemolysin	?	Phagosome maturation	Attenuated ex vivo and in vivo	MAB_2359	69% / 78%
LpdC	Rv0462	SecA2	Lipoamide reductase	Coronin-1	Phagosome maturation	?	MAB_4127c	88% similarity
NdkA	Rv2445c	SecA2	GTPase Activation Protein (GAP)	Rab5, Rab7, Rac1	Phagosome maturation, ROS, apoptosis	Attenuated ex vivo and in vivo (SCID mouse model only)	MAB_1606	75% / 86%
PtpA	Rv2234	?	Phosphatase	VPS33B, Subunit H of V-ATPase, ubiquitin, GSK3	Cytokine response, Phagosome maturation and apoptosis	Attenuated in Guinea pigs, less in mice	MAB_1900c	68% / 81%
PtpB	Rv0153c	?	Phosphatase	?	Apoptosis	Attenuated ex vivo and in vivo (guinea pig)	MAB_4591	44% similarity
Eis	Rv2416c	?	Lysine Nϵ-acetyltransferase activity	JNK	ROS production, autophagy, apoptosis	No attenuation in-vivo	MAB_4532	29% / 44%
SodA	Rv3846	SecA2	Superoxide dismutase	Phagosomal superoxides	ROS production	Attenuated in-vivo	MAB_0118c	82% / 89%
EchA1	Rv0222		Probable enoyl-CoA hydratase	SHP1, TRAF6	Cytokine response	Attenuated in vivo	MAB_0606c	30% / 44%
CpnT/TNT	Rv3903c	?	Hydrolyses NAD+	NAD+	Necrosis	Not attenuated in-vivo	Found in prophages	–
MPT53/DsbE	Rv2878c	Predicted SecA1/2	Disulfide oxidoreductase	Tak1	Triggers Cytokine response	Hypervirulent in-vivo	MAB_3243	63% / 78%

## Author Contributions

MBO wrote the manuscript. MM helped in editing the manuscript. DB edited and conceived the manuscript.

## Funding

MM has a research grant from the Israeli Science Foundation (ISF). DB is supported by NIH R21 grant 1R21AI156415-01A1.

## Conflict of Interest

The authors declare that the research was conducted in the absence of any commercial or financial relationships that could be construed as a potential conflict of interest.

The handling editor declared a past collaboration with the author (DB).

## Publisher’s Note

All claims expressed in this article are solely those of the authors and do not necessarily represent those of their affiliated organizations, or those of the publisher, the editors and the reviewers. Any product that may be evaluated in this article, or claim that may be made by its manufacturer, is not guaranteed or endorsed by the publisher.
